# Rationale, bench testing and *in vivo* evaluation of a novel 5 mm laparoscopic vessel sealing device with homogeneous pressure distribution in long instrument jaws

**DOI:** 10.1186/1750-1164-7-15

**Published:** 2013-12-10

**Authors:** Stefan Eick, Brandon Loudermilk, Erik Walberg, Moritz N Wente

**Affiliations:** 1 Aesculap AG, Am Aesculap-Platz, Tuttlingen, 78532, Germany

**Keywords:** Surgical instruments, Tissue sealing, Laparoscopic surgery, Hemostasis, Electrocoagulation, Electrosurgery, Thermal injury, Burst pressure, Equipment design

## Abstract

**Background:**

In 1998, an electrothermal bipolar vessel sealing (EBVS) system was introduced and quickly became an integral component of the surgical armamentarium in various surgical specialties. Currently available EBVS instruments use a scissor-like jaw configuration and closing mechanism, which causes decreasing compression pressure from the proximal to the distal end of the jaws. A new EBVS system is described here which utilizes a different instrument jaw configuration and closing mechanism to enable a more homogeneous pressure distribution despite longer instrument jaws.

**Methods:**

Results of jaw pressure distribution measurements as well as sealing experiments with subsequent burst pressure measurements *ex vivo* on bovine uterine arteries are demonstrated. Furthermore, an *in vivo* evaluation of the new EBVS system in a canine and porcine model including histological examination is presented.

**Results:**

The device revealed an even pressure distribution throughout the whole jaw length. The *ex vivo* burst pressure measurements revealed high average burst pressures, above 300 mmHg, independent of the outer diameter (1 to 7 mm) of the tested vessels. Histological evaluation of sealed vessels 21 days postoperatively demonstrated sealed and fused vessels without adjacent tissue damage.

**Conclusions:**

The even pressure distribution leading to a sufficient tissue sealing in combination with the novel closing mechanism and extended jaw length differentiates the novel device from other available EBVS systems. This might offer a reduction of the overall procedure time, which should be further evaluated in a clinical study.

## Background

Since the introduction of an electrothermal bipolar vessel sealing (EBVS) system in 1998 and initial reports of its clinical usage, this technology has evolved to a standard method for sealing and transecting blood vessels [1,2]. Today, EBVS systems are offered by various manufacturers and widely used in several surgical specialties such as general and visceral surgery [1,3,4], gynecology [5,6], urology [2,7], cardio-thoracic [8], and pediatric surgery [7,9]. Recent systematic reviews and meta-analyses of randomized controlled clinical trials [10-12] revealed that EBVS systems are safe and at least equally effective concerning blood loss and operating time in comparison to ultrasonic energy devices for blood vessel transection in abdominal surgery.

Besides providing electrothermal energy via a feedback-controlled electrosurgical process, it is equally important for good seal stability and reliable sealing quality to apply sufficient mechanical force to the tissue, as already pointed out in early research articles [13]. Therefore, exact controlling of the compressive force within the instrument is considered as highly important and a well-defined and homogeneous tissue compression throughout the whole length of the instrument jaws is desirable [14,15].

Currently available EBVS instruments use a scissor-like jaw configuration and closing mechanism, which causes decreasing compression pressure from the proximal to the distal end of the jaws. Thus, a possible weakness in the sealing quality can be found at the tip of the instrument, especially when the jaws are completely filled with tissue during the sealing process. The longer the jaws of such a scissor-like instrument are, the more pronounced is this effect. This especially limits the maximum length of the jaws and the sealing electrodes of scissor-like laparoscopic instruments with a diameter of 5 mm.

Because of these effects of uneven pressure distribution and limited electrode length as well as manufacturers focus in the development of the sealing process on speed, surgeons might be accustomed to employing multiple energy activation cycles to seal bigger arteries for a higher confidence in the seal stability. Consecutive sealing procedures next to each other or even at the same position before activating the cutting mechanism of the device is called “double seal” or “overlapping seal”; however, this has only been published rarely in the scientific literature and can sometimes only be evaluated in published videos [16-18].

The aim of the given article is to report on a new EBVS system with focus on a 5 mm laparoscopic vessel sealing instrument, which utilizes a different instrument jaw configuration and closing mechanism to enable a more homogeneous pressure distribution despite longer instrument jaws in comparison to other known vessel sealing instruments. In detail, the jaw configuration and results of jaw pressure distribution measurements as well as sealing experiments with subsequent burst pressure measurements *ex vivo* on bovine uterine arteries are demonstrated. Finally, an *in vivo* evaluation of the new EBVS system in a canine and porcine model including histological examination is presented.

## Methods

### Vessel sealing system

The new EBVS system evaluated in this study is a bipolar electrosurgical system and consists of the Lektrafuse RF generator and the laparoscopic Caiman® 5 instrument (Aesculap AG, Tuttlingen, Germany). The RF Generator is capable of delivering an AC power of 150 W in an impedance range between 10 and 90 Ohms. The instrument-specific feedback-controlled sealing algorithm is primarily optimized for seal quality and safety and is automatically selected, when the instrument is attached to the generator.

**Figure 1:**
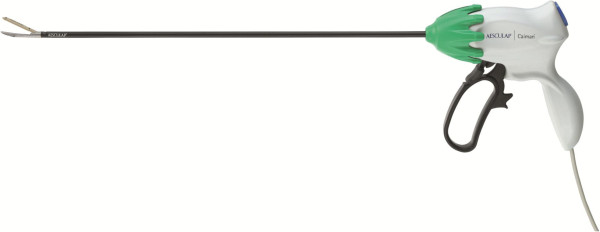
**Caiman**® **5 vessel sealing instrument in a side view.**

The Caiman® 5 is a 5 mm laparoscopic vessel sealing instrument with a shaft length of 36 cm, 360° shaft rotation and a pistol type handle (Figure 1). The instrument jaws carry an upper and a lower sealing electrode. In combination with the Lektrafuse RF generator it can be used to seal blood vessels of up to and including 7 mm diameter and tissue bundles as will fit into the jaws. The sealed blood vessel or tissue can subsequently be divided by an included hand-controlled mechanical blade. The instruments used for all experiments and figures in this publication were prototypes of the first generation of Caiman® 5 instruments, which were exclusively available in the United States.

### Instrument jaws and closing mechanism

**Figure 2:**
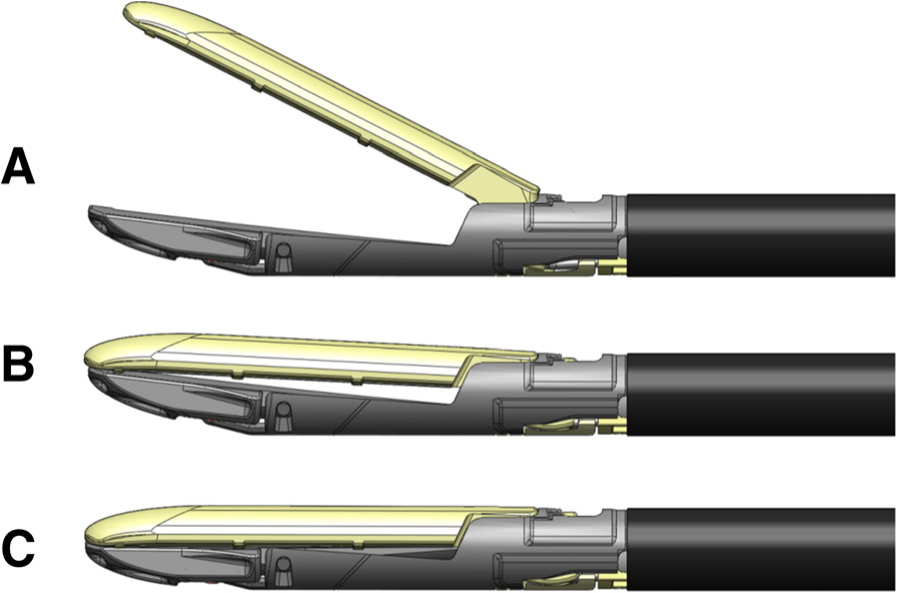
**Schematic side view of the Caiman® 5 hinged jaws closing mechanism.** Jaws open **(A)**, closed at the tip **(B)**, and completely closed **(C)**.

The major novel development in the evaluated EBVS system for a reliable, excellent seal quality is a special hinged jaw design of the instrument. To ensure even pressure distribution and tissue compression in the instrument jaws, an additional hinge at approximately half of the length of the lower jaw is included (Figure 2). The distal pivoting component of the lower jaw, which contains a sealing electrode, is attached at its pivot point to the proximal portion of the lower jaw. The proximal portion of the lower jaw then holds the upper jaw which is fixed to the shaft of the instrument. Thus, the electrode-containing distal portion of the lower jaw is tiltable in relation to the shaft of the instrument as well as to the upper jaw and electrode. A spring is included in the lower jaw assembly, which biases the distal tip towards the upper jaw (Figure 2A). Thus, unlike conventional scissor-like vessel sealing devices, the instrument jaws close first at the distal tip of the instrument (Figure 2B). When further closing the instrument jaws, the movable front part of the lower jaw is able to tilt in relation to the upper jaw in a way that both electrodes are finally aligned in parallel (Figure 2C).

This closing mechanism should avoid that any tissue is being pushed out of the jaws during closing, and enables a parallel jaw alignment with varying thickness of tissue and thus an even pressure distribution and tissue compression within the jaws. In the final configuration, the user enables the sealing process by pushing the activation button one time and the vessels and/or connective tissues are sealed and finally cut mechanically by the surgeon. Due to the closing mechanism and the even tissue compression throughout the whole length of the instrument jaws, the jaws and the sealing electrodes can be longer than in other 5 mm laparoscopic vessel sealing instruments with scissor-like jaws, resulting in a superior sealing length of 26,5 mm.

### Bench testing

#### Jaw pressure measurements

To measure the distribution and amount of pressure within the jaws of the Caiman® 5 instrument, a pressure measurement system (Tekscan, Inc., South Boston, MA, USA) was utilized. For comparison, the jaw pressure was also measured for two conventional vessel sealing instruments: the Covidien LigaSure™ 5 mm and the Covidien LigaSure Advance™ (Covidien Surgical Solutions Group, Boulder, CO). All measurements were made with the same measurement setup and measurement procedure described in this section.

The pressure measurement system consist of a Tekscan 5027 Sensor, which is clamped into the EH-2 Tekscan handle and connected to a personal computer with I-Scan® lite software. The sensor and measurement system were calibrated for two points within the range of force expected (roughly 1.1 MPa) and equilibrated for the study per standard operation procedure and according to manufacturer‘s recommendation.

To simulate the tissue layers, which are compressed within the instrument jaws, a piece (roughly 4 cm by 4 cm) of a 0.5 mm thick compressible silicone pad (50A durometer silicone rubber pad #3788 T21; McMaster-Carr®, Elmhurst, IL) was placed on either side of the pressure sensor. To ensure an accurate force reading, the silicone was placed even with or just inside of the sensors footprint where the jaws are clamped onto the sensor. The sensor with the silicone pads was fully inserted into the jaws of the instrument and the jaw set was closed. The sensor reading was stabilized for approximately two seconds and recorded.

#### Burst pressure measurements

To evaluate the initial sealing quality and reliability of the EBVS system, burst pressure measurements on sealed explanted arteries of various diameters were utilized. The standardized burst pressure measurements were conducted with a laboratory burst pressure measurement system based on a digital gear pump (pump head model #07002-25; gear pump drive model #75211-30; Cole-Parmer, Court Vernon Hills, IL, USA;) as described in the following: the untreated bovine uterine arteries, sized approximately 1 to 7 mm in outer diameter (OD), were freshly frozen directly after harvesting. Before the experiments, the frozen tissue was allowed to thaw to approximately room temperature, bagged and placed in warm water until it had reached approximately 37°C. Testing was performed with tissue temperatures between 25°C (room temperature) and 37°C (body temperature).

Three different size classes of arteries were defined according to their OD when laying approximately flat across a finger: small (1–2 mm), medium (2–4 mm), and large (4–7 mm). The tissue samples were inspected to locate an artery with an OD in the desired size range for sealing and also inspected to assure that the vessels were not cut or damaged in any way. If the artery was encapsulated in connective tissue it was not separated from the tissue.

After confirming the tissue was moist, the artery was clamped with the Caiman® 5 instrument and the sealing was performed by activating the Lektrafuse RF generator once. After completion of the sealing, the arteries were divided using the cutting mechanism on the instrument. Then, the non-sealed end of each artery was slipped on to the appropriate sized dispensing needle attached to the vessel burst pressure measurement system and the artery was secured mechanically onto the needle with hemostats with conformal tubing on each jaw to assure no retrograde leakage. It was mandatory to have at least 5 mm between the seal and the needle.

Burst pressure measurement was started by pumping water into the artery with a flow rate of 15 or 47 ml/min depending on needle diameter. If any leaks were observed along the needle/artery attachment or anywhere along the artery (other than at the sealed end), those leaks were clamped. The maximum pressure was recorded once the artery wall leaked or the sealed end burst. Arteries that were damaged during test set up were not further evaluated.

There is no clear standard given in the literature for a required burst pressure measurement for separating a successful artery seal from a failure. Published values strongly vary from 50 mmHg [19], 150 mmHg [8], 250 mmHg [14], 300 mmHg [20-22], 360 mmHg [23], up to 400 mmHg [24]. Nevertheless, burst pressure provides a reproducible stability index of the seal. We chose 300 mmHg or 2.5 times of the average systolic artery pressure as requirement for a successful seal, suggesting this will be safe under systolic pressure in normal patients or those with treated hypertension.

### Animal test

Animal tests with blood vessel sealing were performed to prove the sufficiency of the vessel sealing system’s capabilities *in vivo* as well as to evaluate acute and chronic survival of the animals. Furthermore, the sealed tissues were evaluated macroscopically and histologically.

The animal studies were designed as regulatory study required by the FDA for device clearance and were performed at BioSurg, Inc. animal facility (Winters, CA, USA). BioSurg is a certified Class R Research Facility under the Animal Welfare Act (7 U.S.C. 2131 et seq., Certificate No. 93-R-0200, Customer No. 1139) and maintains an Animal Welfare Assurance (No. A3751-01) by the Office of Laboratory Animal Welfare (OLAW) that was submitted in compliance with the Public Health Service (PHS) Policy on Humane Care and Use of Laboratory Animals. The Institutional Animal Care and Use Committee (IACUC) reviewed and approved all activities involving the use of live "covered" animals within this study prior to their initiation (Protocol No. SA0908n).

Overall, the experiments were performed on three dogs and one pig. The Caiman® 5 was used to seal and divide mesenteric and parametrial tissues and structures surrounding the targeted organs. For every transaction site, the seal process was only activated once. To demonstrate substantial equivalence, another dog was operated on using the predicate, conventional EBVS system (LigaSure™ 5 mm instrument and ForceTriad™ Energy Platform, Covidien). The animals were examined acutely for vessel sealing and were then survived chronically for 21 days. Each animal was positioned in dorsal recumbency, anesthetized and surgically prepped and draped according to BioSurg’s standard operating procedures. A BioSurg Veterinary surgeon performed the operative procedures using sterile technique throughout all procedures.

In the three dogs, various surgical procedures including left nephrectomy, ovariohysterectomy and splenectomy were performed with the Caiman® 5. In the comparative dog, a splenectomy and nephrectomy were performed with the conventional EBVS system. In the pig, the left carotid artery was transected using the Caiman® 5. Several treated vascular segments were removed acutely and preserved in 10% Neutral Buffered Formalin (NBF) for histologic analysis at a later date. After the sealed tissues had been examined for adequate hemostasis, the abdomen was closed and each animal recovered as per BioSurg’s anesthesia and surgical SOPs. The animals were survived post operatively for a period of 21 days.

### Histology/pathology

After 21 days, the animals were sacrificed and a necropsy was performed on each animal with emphasis on identifying the treatment sites. During necropsy, evidence of mechanical or thermal injury to adjacent tissue structures and recent or active bleeding from treatment sites was assessed. Chronic treatment sites were photographed, excised *en bloc*, and immersion fixed in 10% NBF. All transected vascular segments (acute and chronic) were trimmed and routinely processed for histopathologic processing and microscopic analysis. Briefly, tissues were dehydrated in a series of graded alcohol solutions, cleared in xylene, embedded in paraffin, sectioned at 4–6 microns, and stained with hematoxylin and eosin (H&E) for light microscopic evaluation. Acute and chronic histology were compared to assess efficacy/safety concerns that could be related to vessel leakage at the occlusion site due to thermal injury and possible tissue necrosis.

## Results

### Bench testing

#### Jaw pressure distribution measurement

**Figure 3:**
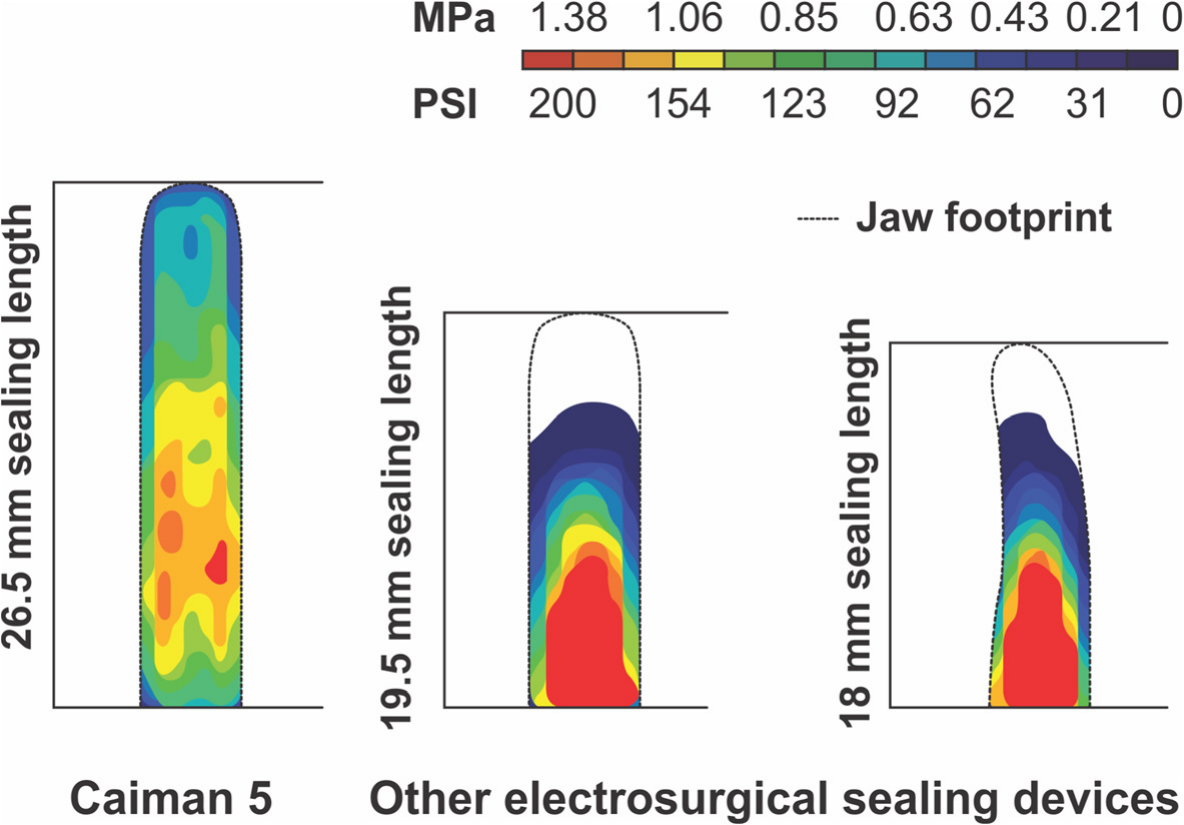
**Jaw length and compression pressure distribution.** The compression within the jaws of the Caiman® 5 sealing device in comparison to other electrosurgical sealing devices with scissor-closing jaws is shown. For the conventional devices, the pressure decreases from the proximal end of the jaws to the distal tip, reaching a pressure of virtually zero within the last quarter of the jaw length. The hinged jaw of the new device enables much more even pressure distribution throughout the jaw length of 26,5 mm. Pressure data outside of the jaw footprint (dashed line) was removed for clarity.

The results of jaw pressure measurements of the Caiman® 5 instrument with the hinged jaw design and two conventional vessel sealing instruments with scissor-like jaws are shown in Figure 3.

While the hinged jaw of the Caiman® 5 enables an even pressure distribution in a range between roughly 0.6 and 1.2 MPa throughout the whole jaw length with only a slight medial maximum, the other two sealing devices show an uneven pressure distribution. The pressure strongly decreases from more than 1.4 MPa at the proximal jaw end to virtually zero at the distal tip and within the last quarter of the jaw length. Figure 3 also shows the jaws and the sealing electrodes of the Caiman® 5 are longer in comparison to the jaws and electrodes of the conventional sealing devices.

#### Burst pressure measurements

Table 1 summarizes the results of 272 *ex vivo* burst pressure measurements done with the Caiman® 5 instruments on unfrozen bovine uterine arteries with outer diameters between 1 and 7 mm. As a requirement for a successful seal, a minimum burst pressure of 300 mmHg, 2.5 times standard systolic arterial blood pressure, was chosen.

**Table 1:** Burst pressure measurements

	** OD small (n = 85)**		** OD medium (n = 92)**		** OD large (n = 95)**	
	**OD (mm)**	**BP (mmHg)**	**OD (mm)**	**BP (mmHg)**	**OD (mm)**	**BP (mmHg)**
**Mean ± SD**	1.9 ± 0.3	1015 ± 536	3.3 ± 0.6	1017 ± 514	5.2 ± 0.9	865 ± 389
**Min / max**	1.0 / 2.0	131 / 2975	2.0 / 4.0	304 / 2428	4.0 / 7.0	313 / 2288
**BP < 300 (n)**	1		0		0	

Results of burst pressure (BP) measurements for arteries with varying outer diameters (OD) between 1 and 7 mm. For all values, the mean, standard deviation (SD), minimum, and maximum values are shown. OD small: 1–2 mm, OD medium: 2–4 mm, OD large: 4–7 mm.

For all three size classes of arteries (OD small, OD medium, and OD large), the burst pressure measurements showed high average burst pressures of roughly 3 times the minimum requirement. Out of all 272 measurements, only for one artery (size OD small, burst pressure 131 mmHg), the minimum burst pressure was not reached.

### Animal test

Altogether, 70 blood vessel and tissue seals, including 38 arteries with outer diameters between 1 and 8 mm (3.0 ± 2.3 mm; mean ± SD) and 28 non-arterial vessels with outer diameters between 1 and 10 mm (3.0 ± 2.5 mm), were performed within the three dogs and one pig. Each site was transected with a single activation of the sealing process and subsequent cutting.

For all 70 seals, no acute seal failure was observed. The average seal duration was 3.9 ± 1.1 s (min: 1.8 s, max: 6.4 s). The average measured thermal spread beyond the instrument jaws was less than 1 mm. No arcing and charring and only minor tissue adhesion occurred. All four animals survived for 21 days with no postoperative complications and acceptable animal stress. After 21 days, the reoperation (necropsy) was performed, showing no chronic seal failures and no mechanical or thermal injury in the treatment sites for all animals.

### Histology/pathology

#### Canine model

**Figure 4:**
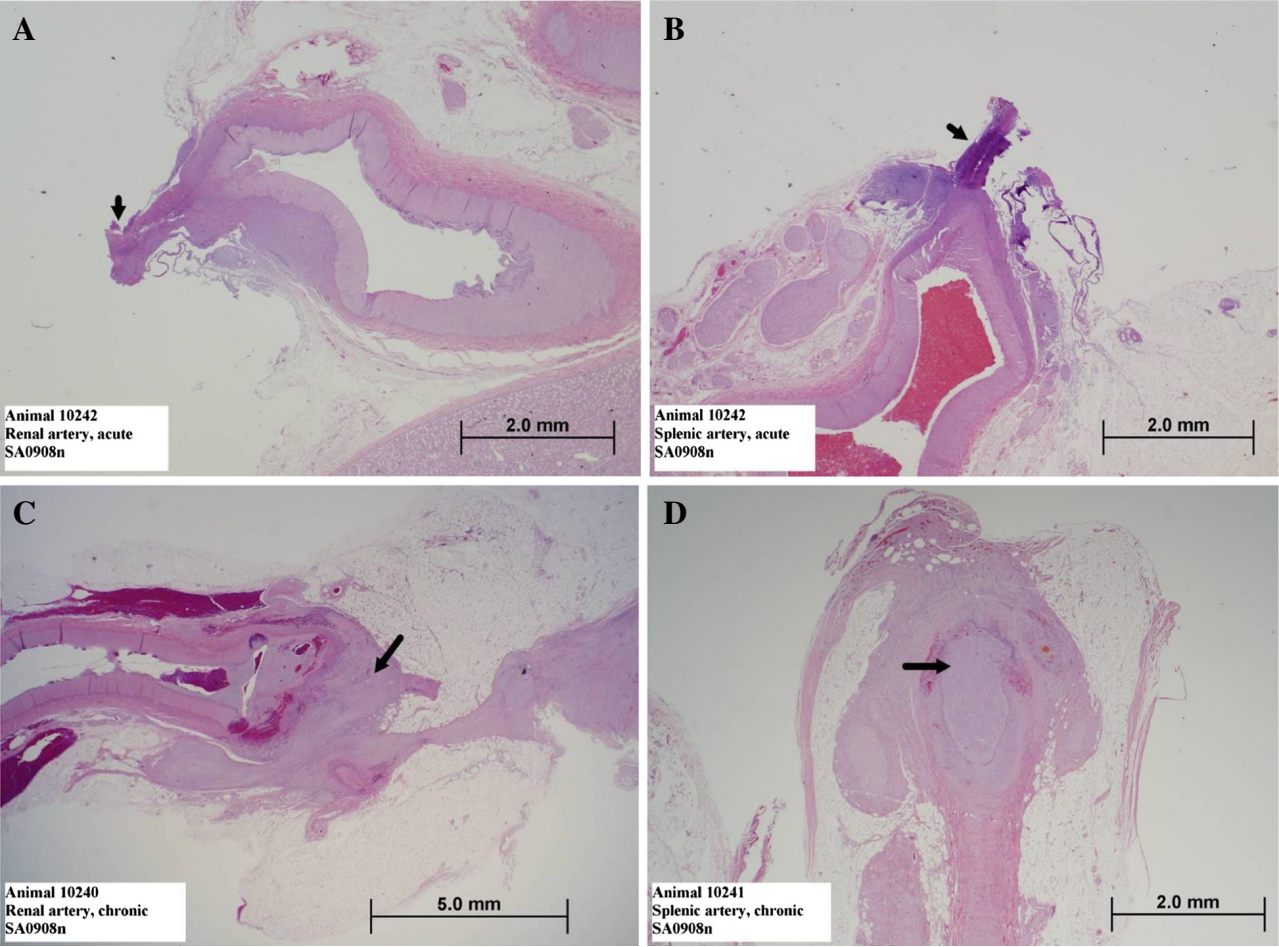
**Histological evaluation of arteries sealed with the Caiman**® **5 instrument.** Photomicrographs of H&E stained sections in acute **(A, B)** and chronic phase **(C, D)** after sealing with the Caiman® 5 instrument. Serial sections through renal **(A, C)** and splenic **(B, D)** arteries. **A, B:** For both chronic arteries, the black arrow demonstrates the transected end of the artery, which is characterized by coagulative thermal necrosis. The tissue appears fused and sealed. C: The black arrow demarcates the distal end of the transected segment surrounded by a cap of fibroplasia. There is an occlusive, organized luminal thrombus, and the distal end of the artery is suffused with hemorrhage; a modest amount of fibrous fibers dissects into the surrounding fat. D: The black arrow indicates the occlusive organized luminal thrombus. The transected end of the artery is surrounded by a fibrous cap.

All acutely collected arteries of the three dogs demonstrated similar histopathologic changes. The transected ends of the arteries revealed fresh coagulative thermal necrosis and appeared sealed and fused. In the more distal sections of the arterial segments, a disruption of the intimal layer was noted. There was no further adjacent tissue damage. As representative examples, Figure 4 shows photomicrographs of H&E stained acute sections through a renal (A) and a splenic (B) artery.

After 21 days, gross assessment showed healed treatment sites with no indication of recent or active bleeding and of adjacent tissue or organ damage. Microscopically, the treated arterial segments all demonstrated occlusive thrombosis at the transected ends with a surrounding cap of organized fibrotic tissue. The luminal thrombus was typically organized with sprouting of endothelial cells and fibroblasts forming interconnected microvascular channels and focally lympho-histiocytic inflammatory cells. Figure 4 (C,D) shows representative photomicrographs of H&E sections stained in “chronic stage” as examples.

The remaining transected ends of the arteries demonstrated both coagulative thermal necrosis (a result of the initial treatment) and lytic necrotic change (a result of the subsequent inflammatory process). The surrounding fibrous cap was minimally inflamed with lymphocytes and histiocytic cells. Xanthogranulomatous changes, often with scant mineralized centers, were also scattered in the vicinity in the surrounding adherent fat and nerve bundles.

All acute and chronic histopathologic tissue changes were similar to those found in the comparative fourth dog, which was operated on with the conventional blood vessel sealing system.

#### Porcine model

The transection site of the carotid artery was identified grossly and was surrounded by fibrous adhesion. There was no evidence of recent or on-going bleeding. Proximal and distal sections of the carotid artery were evaluated microscopically.

Similar to the canine samples, there was an occlusive luminal thrombus in both segments, which was organized by fibroblasts and contained pockets of fibrin or blood. Organized fibrovascular tissue capped both the proximal and distal ends. A minimal degree of lymphohistiocytic inflammation was present throughout.

## Discussion

Strongly driven by the increasing percentage of minimally invasive surgical procedures, more advanced electrosurgical instruments with the ability to transect tissues and seal vessels became a standard in various fields of surgery, since their introduction just more than a decade ago. The instruments undergo a steady development, as surgeons demand them for more and more complex procedures, such as liver and pancreatic resections [25,26].

In particular an increased jaw length with equally distributed closing pressures and a safe closing mechanism starting at the tip of the device were the demands when the development of the new device started. Due to the laws of mechanics, every scissor-like instrument produces an uneven pressure distribution between the jaws when grasping tissue. The uneven pressure distribution is more pronounced as the tissue thickness between the jaws is increased. For blood vessel sealing instruments, it is well known that sealing process and sealing quality strongly depend on the pressure [14,15]. Therefore, every scissor-like sealing instrument produces uneven sealing quality due to strong pressure variations from the proximal to the distal end of the jaws. Especially at the tip of the instrument, a satisfactory sealing quality cannot be guaranteed, when the jaw of the instrument is completely filled with tissue. Due to the hinged jaw, the Caiman® 5 instrument shows an even pressure distribution with only a slight maximum at the middle of the jaw length, as could be shown with pressure measurements in the instrument jaws.

Because of the even pressure distribution in hinged jaws, it was possible to construct the Caiman® 5 instrument with long jaws with 26,5 mm of sealing length, which is one third longer than other available vessel sealing instruments on the market. This allows the sealing of more tissue with one bite than with other instruments. In combination with an optimized sealing process, the instrument allows for constant sealing quality throughout the whole length of the instrument jaws with a single activation of the sealing process, as was shown with successful sealing experiments both *ex vivo* and *in vivo*.

On explanted bovine arteries, the burst pressure measurements showed reliable sealing results with average burst pressures of roughly three times the minimum requirement for all three evaluated size classes of arteries. Those results are in the same range or even better than reported values in the literature [8,14,15,19-24] and demonstrate the reproducibility of a high initial stability of artery seals with the new EBVS system using the Caiman® 5 instrument.

In a canine and a porcine animal model, the acute and chronic stability and healing of artery seals with the novel instrument was evaluated and compared to seals with a conventional system *in vivo*. The gross and histological findings were similar in all animals at each site. There was no evidence of recent or active bleeding at any of the transection sites, including the left carotid artery in the porcine animal model, showing reliability of the Caiman® 5 instrument even for sealing of large blood vessels.

## Conclusions

Quality and reliability of blood vessel sealing strongly depends on the pressure in the instrument’s jaws. Scissor-like instruments show strong pressure variations from the proximal to the distal end of the jaws, potentially leading to uneven sealing quality with possible weaknesses at the tip of the instrument. Utilizing an additional hinge in the lower instrument jaw, the Caiman® 5 instrument establishes an even pressure distribution within the instrument’s jaws, which are longer than other 5 mm laparoscopic blood vessel sealing instruments’ jaws. As shown *ex vivo* and *in vivo*, the system enables acute seal stability with a single process activation and chronic healing of blood vessel transaction sites. The evaluated EBVS system offers a constant level of confidence in seal quality (safe hemostasis with one seal) in the tested setup and, in combination with the longer instrument jaws, the prospective of a reduced overall procedure duration, which should be evaluated in a clinical trial.

## Competing interests

SE, BL, EW and MNW are full-time employees of Aesculap AG, Germany. BL and EW are involved in patent applications of the described newly developed surgical instruments. All authors declare that they have no further competing interests.

## Authors’ contributions

SE performed the literature review, SE and MNW drafted the manuscript and made substantial contributions to manuscript conception and interpretation of data. BL and EW devised the discussed instrument, strongly contributed in acquisition, analysis, and interpretation of data, and revised the manuscript. All authors read and approved the final manuscript.
